# PSYCHOSOCIAL CHARACTERISTICS BY PAIN PRESENCE AND LIMITATIONS AMONG OLDER ADULTS

**DOI:** 10.1093/geroni/igad104.2445

**Published:** 2023-12-21

**Authors:** Ashleigh Holmes, Weijun Wang, Yu-Ping Chang

**Affiliations:** University at Buffalo, The State University of New York, Buffalo, New York, United States; University at Buffalo, The State University of New York, Buffalo, New York, United States; University at Buffalo, The State University of New York, Buffalo, New York, United States

## Abstract

Older adults experience high rates of pain, which is linked to physical disability, poorer mental health, reduced social engagement, cognitive impairment, and increased health care costs. Due to the heterogeneous nature of pain, further research is needed to identify psychosocial characteristics in older adults grouped by pain experience. Using the nationally representative National Health and Aging Trends Study Round 11 (2021) dataset, we performed ten multiple regression models (controlling for age, gender, and race/ethnicity) examining various psychosocial variables in 3,381 older adults divided into three pain groups: no pain within the last month, pain without activity limitations, and activity-limiting pain. The results showed that older adults with activity-limiting pain compared to those without pain had significantly higher depression (b=0.724, p<.001), anxiety (b=0.675, p<.001), fear of falling (b=1.078, p<.001), difficulty initiating and maintaining sleep (b=0.906, p<.001), and use of sleep medications (b=0.448, p<.001), as well as reduced positive affect (b=-1.812, p<.001), self-realization (b=-0.204, p<.001), self-efficacy (b=-0.041, p=.012), resilience (b=-0.101, p<.001), and social participation (b=-0.205, p<.001). In older adults with non-activity-limiting pain compared to those without pain, all relationships remained significant except for self-realization (p=.366), self-efficacy (p=.122) and resilience (p=.123). Furthermore, older adults with non-activity-limiting pain reported higher levels of self-realization (b=0.173, p<.001) and self-efficacy (b=0.068, p<.001) than those with activity-limiting pain. The findings suggest that pain is strongly associated with all psychosocial outcomes, especially in older adults with activity-limiting pain. Future research should examine the impact of self-realization and self-efficacy on pain experiences in older adults with activity-limiting pain.

